# Reply to: Problems with two recent Petri net analyses of Neanderthal adhesive technology

**DOI:** 10.1038/s41598-024-60674-7

**Published:** 2024-05-07

**Authors:** Sebastian Fajardo, Paul R. B. Kozowyk, Geeske H. J. Langejans

**Affiliations:** 1 Department of Materials Science and Engineering, Delft University of Technology, Delft, Zuid-Holland 2628CD The Netherlands; 2 Palaeo-Research Institute, University of Johannesburg, Johannesburg, Gauteng 2092 South Africa; 3 Leiden Institute of Advanced Computer Science (LIACS), Leiden University, Leiden, Zuid-Holland 2333CC The Netherlands

**Keywords:** Archaeology, Computer science

**replying to**: P. Schmidt; *Scientific Reports* 10.1038/s41598-024-60793-1 (2024).

## Introduction

In two recent publications^1,2^ we introduce Petri nets as a new method to model ancient technological systems and their complexity. We use previously proposed methods of prehistoric tar production as case studies and apply three different metrics that each rely on unique definitions of complexity: (1) The density metric considers the interconnectedness between events and resources and can be related to requirements of simultaneous information processing; (2) The extended cyclomatic metric concerns the likelihood of errors throughout the process, and the potential need for planning and inhibition control; (3) The structuredness metric relates to the effort to understand abstract information about the materials, product templates and the process itself, and thus to learning. The results can therefore be interpreted along behavioural and cognitive lines. Our application of Petri net modelling to different tar production methods demonstrates that there is much variation in complexity between tar technologies. Moreover, we can indicate where these differences stem from. This is relevant to debates where technology is a proxy for cognition. Schmidt and Tennie^3^ misinterpret our work. They claim it is subjective, and not rooted in reality. Here we take the opportunity to address any misinterpretation.

## Experiments, the archaeological record, and reality

Schmidt and Tennie^3^ highlight elements in our models that the authors consider subjective, or things that are automatic occurrences. The latter implying that these elements do not faithfully represent the organisation of technology. This shows a misunderstanding of the method. Unlike *chaîne opératoire* analysis, Petri nets are not a sequential representation of human actions, but a causal model of a system. As explained in our papers (p. 4^1^) we incorporated events that changed the location or modified the physical properties of resources. Because of the formal nature of the method, our modelling decisions are made explicit and are described. Moreover, Petri nets provide a framework to implement robustness tests (p. 4–5^1^). We found limited differences in the granularity of the modelled events. Therefore, the atomic unit size does not affect our conclusions. This means that the method is systematic and our results reproducible.

Our models are representations of the mechanisms in real-world systems, enabling us to comprehend specific aspects of these systems. But models are of course not reality, and we do not claim they are. Where Schmidt and Tennie^4^ rely only on experiments, we expand with in-silico modelling of experimental data. Experimental designs and empirical data are fundamental to understanding the evolution of cognition and culture. However, causal models like ours facilitate comparisons of the external validity of various individual studies or experiments; continually expanding the models and incorporating more diverse experiments will improve this new method.

Schmidt and Tennie^3^ suggest that one tar production method, condensation, is not evidenced in the archaeological record. We consider condensation a likely early method for tar production (as Schmidt and Tennie have also suggested^4^). However, because the tar yield from condensation is limited, scaling was employed by Blessing and Schmidt^5^ to make this method more comparable with other techniques. In one of our papers^2^, we explore what scaling does to system complexity. We appreciate the opportunity to clear up a misunderstanding here. We do not argue in favour of a specific tar production method nor believe that any were beyond the cognitive capabilities of Neanderthals, as suggested by Schmidt and Tennie^3^. We show that three concurrent condensation processes increase complexity, something that was not addressed previously^5^. This increase in complexity from scaling, and the generous application of birch tar on Palaeolithic tools^6^, raises the probability that more economical solutions to increase tar yield were found.

The concerns raised by Schmidt and Tennie^3^ regarding potential errors in three cobble condensation may stem from the use of different definitions for concepts like error, anticipation, risks and decisions. The authors appear to confuse our models with other approaches that use sequential models like *chaîne opératoires* and cognigrams^7–9^ to map human behaviour. In Petri nets, the causality between local events in a process is recorded, and subsequently metrics can be applied to assess different aspects of the process. The likelihood of events like ‘relighting bark’ (highlighted by Schmidt and Tennie^3^) occurring causes an increase in the complexity. In analysing the scalability problem^2^, we use the much-increased reachability graph of the upscaled method to show that there are more decisions made by the operator in this process. With every decision there is a chance for an error. This does not mean that no tar is produced; this means that when you look at the model through a probability lens there is a higher chance of partially satisfying the intended aim of the process. Counterintuitively, the upscaled method has a sharp increase in the cyclomatic metric score^2^ compared to the one-cobble method. With every added possibility to the system, the metric is affected exponentially.

## Returning to logical fallacies

Schmidt and Tennie^3^ treat an individual operator’s recollections as definitive, falling into the unavoidable subjectivity of the human experience. Their video evidence succeeds in demonstrating fully modern human levels of response inhibition, multi-tasking, and task switching (Supplementary video 2^5^). Further, the operator is clearly engaged in near constant monitoring and decision-making, suggesting reliance on the brain’s processing power. We agree the operator is not overwhelmed by the complexity of the process, but we cannot a priori assume people in the deep-past solved the problem of how to make tar in the same way.

Video data from Schmidt et al. shows that during a three-cobble condensation experiment, the operator switches tasks nearly twice as frequently as when using a single cobble (approximately every 5 seconds^5^ vs. 9 seconds^4^, respectively). Given that cognitive complexity can be defined as the capacity for task switching and response inhibition, among other aspects^10^, these observational details are also worth considering, rather than relying on the perceived difficulty from the experimenter. The three-cobble video also shows tar scraping being interrupted to ignite and re-arrange bark on other stones; something which is not necessary in the one-cobble process. In addition, the authors state tar burning away was not observed^3^, but to our knowledge they never measured this. In our experiments^1,2,11^, more frequent scraping resulted in higher yields of tar, suggesting that leaving bark burning longer reduces efficiency. Regardless of these details, such videos are a valuable resource for fine-tuning future Petri net models and testing them against natural behaviour.

## Another tool for the toolbox

The suitability of different methods to analyse prehistoric technological organisation is indeed debatable. We argue that Petri nets are promising because they can highlight different facets of process complexity and by proxy different aspects of cognition. Moreover, Petri nets are a useful tool for synthesizing concurrent production systems from sequential observations. This applies particularly well to processes like the creation of birch tar or compound technologies. Additionally, developing new folding algorithms on different Petri net models of the same production process can facilitate the identification of the essential elements in production processes. Schmidt and Tennie^3^ suggest that their toolkit is better suited to identifying the advent of physical and cultural changes. Their method serves specific research questions, and therefore incorporates specific variables and empirical datasets. For example, ‘difficulty’, measured as success rate of modern human subjects, and ‘time’. We find these variables less useful because we are asking different questions.

We see our approach and other methods like cognigrams and procedural units^8,12–15^, as a way to further ideas posed by other researchers. For example, when selecting the metrics to examine our models^1^, we drew from Wadley’s and others’ proxies to identify complex cognition in technology^10,16,17^. Our aim^1^ was partly to quantify these traits. In addition, rather than simply pinpointing when a technological change took place, we are interested in asking how differing technologies, e.g. making bow and arrows, pottery, and distilling tar, can be quantitatively compared in terms of the complexity of their organisation. Petri nets can also provide alternative definitions for the author’s ‘difficulty’, making this model more applicable to researchers with different questions. Finally, system complexity should be measured in multiple ways, as Petri nets can do with a wide range of metrics and definitions, doing justice to the diversity of approaches hominins use to overcome technological problems.

## Conclusion

Petri nets can accommodate a wide array of research questions in archaeology, with the benefit of being explicit and transparent. Although experimental archeology leads to a fundamental corpus of data on transformative technologies, these data alone do not provide answers to all questions about the past. Looking forward, larger and more diverse datasets can feed and refine in-silico models like ours to answer questions that are difficult to explore with individual experimental designs. In that light, we are happy to see that recent experimental and archaeological work by Schmidt and Tennie^18^ confirms our theoretical expectations about the evolution of technology.
